# Prediction of Tumor Shrinkage Pattern to Neoadjuvant Chemotherapy Using a Multiparametric MRI-Based Machine Learning Model in Patients With Breast Cancer

**DOI:** 10.3389/fbioe.2021.662749

**Published:** 2021-07-06

**Authors:** Yuhong Huang, Wenben Chen, Xiaoling Zhang, Shaofu He, Nan Shao, Huijuan Shi, Zhenzhe Lin, Xueting Wu, Tongkeng Li, Haotian Lin, Ying Lin

**Affiliations:** ^1^Breast Disease Center, The First Affiliated Hospital, Sun Yat-sen University, Guangzhou, China; ^2^State Key Laboratory of Ophthalmology, Zhongshan Ophthalmic Center, Sun Yat-sen University, Guangzhou, China; ^3^Department of Radiology, The First Affiliated Hospital, Sun Yat-sen University, Guangzhou, China; ^4^Department of Pathology, The First Affiliated Hospital, Sun Yat-sen University, Guangzhou, China; ^5^Zhongshan School of Medicine, Sun Yat-sen University, Guangzhou, China; ^6^Center for Precision Medicine, Sun Yat-sen University, Guangzhou, China

**Keywords:** breast cancer, multi-parametric MRI, neoadjuvant chemotherapy, radiomics, machine learning, tumor shrinkage pattern

## Abstract

**Aim:** After neoadjuvant chemotherapy (NACT), tumor shrinkage pattern is a more reasonable outcome to decide a possible breast-conserving surgery (BCS) than pathological complete response (pCR). The aim of this article was to establish a machine learning model combining radiomics features from multiparametric MRI (mpMRI) and clinicopathologic characteristics, for early prediction of tumor shrinkage pattern prior to NACT in breast cancer.

**Materials and Methods:** This study included 199 patients with breast cancer who successfully completed NACT and underwent following breast surgery. For each patient, 4,198 radiomics features were extracted from the segmented 3D regions of interest (ROI) in mpMRI sequences such as T1-weighted dynamic contrast-enhanced imaging (T1-DCE), fat-suppressed T2-weighted imaging (T2WI), and apparent diffusion coefficient (ADC) map. The feature selection and supervised machine learning algorithms were used to identify the predictors correlated with tumor shrinkage pattern as follows: (1) reducing the feature dimension by using ANOVA and the least absolute shrinkage and selection operator (LASSO) with 10-fold cross-validation, (2) splitting the dataset into a training dataset and testing dataset, and constructing prediction models using 12 classification algorithms, and (3) assessing the model performance through an area under the curve (AUC), accuracy, sensitivity, and specificity. We also compared the most discriminative model in different molecular subtypes of breast cancer.

**Results:** The Multilayer Perception (MLP) neural network achieved higher AUC and accuracy than other classifiers. The radiomics model achieved a mean AUC of 0.975 (accuracy = 0.912) on the training dataset and 0.900 (accuracy = 0.828) on the testing dataset with 30-round 6-fold cross-validation. When incorporating clinicopathologic characteristics, the mean AUC was 0.985 (accuracy = 0.930) on the training dataset and 0.939 (accuracy = 0.870) on the testing dataset. The model further achieved good AUC on the testing dataset with 30-round 5-fold cross-validation in three molecular subtypes of breast cancer as following: (1) HR+/HER2–: 0.901 (accuracy = 0.816), (2) HER2+: 0.940 (accuracy = 0.865), and (3) TN: 0.837 (accuracy = 0.811).

**Conclusions:** It is feasible that our machine learning model combining radiomics features and clinical characteristics could provide a potential tool to predict tumor shrinkage patterns prior to NACT. Our prediction model will be valuable in guiding NACT and surgical treatment in breast cancer.

## Introduction

Neoadjuvant chemotherapy (NACT) has been used as the standard treatment to downstage tumor in inoperable patients with locally advanced breast cancer, while for operable patients, it is increasingly being used to reduce tumor size and increase the possibility of breast-conserving surgery (BCS) (Hennessy et al., 2005; Mathew et al., 2009; Mougalian et al., 2016). The 2017 St. Gallen International Expert Consensus Conference showed that NACT had been extensively used in patients with human epidermal growth factor receptor 2 positive (HER2+) and triple-negative (TN) breast cancer, especially those with axillary lymph node metastasis, to improve survivals (Curigliano et al., 2019). Pathological complete response (pCR), which is defined as ypT0/is after NACT according to the *American Joint Committee on Cancer (AJCC) TNM Staging Manual*, 8th Edition, has been proven as a good prognostic marker to predict a successful long-term survival in breast cancer (Kong et al., 2011; Giuliano et al., 2018). But only about 30% of the patients achieved pCR after NACT, and the pCR rate varied in different molecular subtypes, as tumor size and treatment regimen influence the treatment response (Chen et al., 2014; Cortazar et al., 2014; Goorts et al., 2017).

After NACT, breast cancer shows different shrinkage patterns as follows: (a) no residual tumor, (b) no invasive tumor but residual ductal carcinoma in situs (DCIS), (c) concentric shrinkage, (d) a main residual invasive focus with surrounding DCIS, (e) multicentric shrinkage (i.e., more than two invasive lesions), (f) stable disease (SD), and (g) progressive disease (PD). The former two patterns are considered as pCR after NACT while pCR and concentric shrinkage are both considered as sufficient tumor responses which can benefit from BCS, and the negative surgical margins are easier to achieve for them. The mechanism of how some biological factors such as tumor subtypes influence tumor shrinkage pattern is still unclear. Earlier studies showed that HER2+ and TN breast cancer had a higher possibility to achieve a sufficient tumor response than hormone receptor positive (HR+) but HER2– breast cancer after NACT. Breast pCR is rarely achieved in HR+/HER2– breast cancer due to the low chemosensitivity, but such molecular subtype of cancer can also benefit from BCS (Ballesio et al., 2017; Eom et al., 2017). The assessment of pCR is insufficient to determine patients suitable for BCS since tumor concentric shrinkage is also suitable. Hence, the tumor shrinkage pattern is a more reasonable marker than pCR to choose candidates for BCS. Another study has shown that about 10–35% of patients had a poor response to NACT (SD/PD), which indicates a high risk of local recurrence after surgery (Li et al., 2020). For these patients, it is imperative to avoid the associated adverse toxicity of chemo-drug and overtreatment.

In order to identify the patients who have a sufficient response to NACT and can benefit from BCS, it is essential to predict tumor shrinkage pattern prior to treatment. Lobbes et al. revealed that magnetic resonance imaging (MRI) has better accuracy in assessing residual tumor after NACT than physical examination, mammography, and ultrasonography in patients with breast cancer (Lobbes et al., 2013). The ACRIN 6657/I-SPY Trial has reported that MRI in the early stage of NACT could provide much helpful information about tumor pathological response (Hylton et al., 2012). Some studies have revealed that dynamic contrast-enhanced imaging (DCE) could distinguish residual tumor from therapy-induced non-vascularized fibrosis (Pickles et al., 2005; Manton et al., 2006; Padhani et al., 2006; Loo et al., 2008). The pre-NACT MRI can be used to assess the extent and morphology of primary breast cancer and may provide useful information about tumor shrinkage patterns. However, a meta-analysis reported that the MRI data had limited value for the prediction of pCR with the sensitivity of 64% in breast cancer (Yuan et al., 2010).

Radiomics is a frontier interdiscipline of medical imaging and computer field, and it has been used to extract much quantitative information from medical images (Lambin et al., 2012; Aerts et al., 2014; Yip and Aerts, 2016). The radiomics information has shown a great potential to assist clinicians, and several radiomics models had been constructed to better diagnose disease and monitor tumor response to treatment in breast cancer (Antunovic et al., 2019; Li et al., 2020; Zheng et al., 2020; Zhuang et al., 2020). Liu et al. developed a radiomics model for predicting pCR after NACT in breast cancer based on mpMRI and validated the model by multicenter datasets with the AUCs of 0.71–0.80 (Liu et al., 2019). Radiomics can help acquire more information from MRI to better predict tumor shrinkage patterns prior to NACT. However, to our knowledge, the feasibility of radiomics to predict tumor shrinkage pattern based on mpMRI and clinicopathologic characteristics prior to NACT still remains to be tested, and no study investigated the correlation between the tumor shrinkage pattern and the mpMRI radiomics features in different molecular subtypes of breast cancer using the machine learning method. Therefore, the purpose of our study is to explore the radiomics biomarkers of tumor shrinkage pattern from mpMRI, construct a prediction model combined with the clinicopathologic characteristics, and investigate the predictor based on the molecular subtype of breast cancer.

## Materials and Methods

### Study Population

We retrieved 503 consecutive patients with breast cancer who were treated with NACT and followed by surgery in our center between March 2016 and July 2020. The inclusion criteria for this study were as follows: (1) the patient had a biopsy-proven unilateral breast cancer, (2) the patient successfully completed NACT and following breast surgery in our center, (3) the MRI examination of the breast was performed before the initiation of NACT in our hospital within 2 weeks, and (4) the baseline data were complete. The exclusion criteria were as follows: (1) the patient had prior treatment to breast cancer, (2) the pathological results or clinical data were unavailable, (3) the patient did not complete standard NACT, or the surgery was not performed in our center, (4) the MRI data were unavailable, or imaging quality was insufficient, and (5) the patient had a metastatic disease or other malignance. Finally, a total of 199 patients met the criteria. The clinicopathologic characteristics of each patient including age, menstrual state, clinical anatomical TNM staging according to the *AJCC Manual*, 8th Edition, and the pathological biopsy results including tumor type, receptor status, and tumor proliferation rate (i.e., Ki-67 index) were derived from the electronic medical records. The characteristics of all patients are summarized in Table 1.

**Table 1:** Clinical and histopathological characteristics of study population grouped by tumor shrinkage pattern.

**Characteristics**	**Total patients (*n* = 199)**	**Type 1 shrinkage (*n* = 105)**	**Type 2 shrinkage (*n* = 94)**	***p*-value**
Age (mean ± SD)	46.85 ± 10.13	47.95 ± 10.19	45.62 ± 9.97	0.105
Menopausal status				0.819
Premenopausal	116 (58.3%)	62 (59.0%)	54 (57.4%)	
Postmenopausal	83 (41.7%)	43 (41.0%)	40 (42.6%)	
Histology				0.152
IDC	191 (96.0%)	103 (98.1%)	88 (93.6%)	
Other	8 (4.0%)	2 (1.9%)	6 (6.4%)	
Clinical stage				0.133
II	98 (49.2%)	57 (54.3%)	41 (43.6%)	
III	101 (50.8%)	48 (45.7%)	53 (56.4%)	
Clinical T stage				0.139
1	5 (2.5%)	3 (2.9%)	2 (2.1%)	
2	127 (63.8%)	73 (69.5%)	54 (57.4%)	
3	50 (25.1%)	24 (22.9%)	26 (27.7%)	
4	17 (8.6%)	5 (4.7%)	12 (12.8%)	
Clinical N stage				0.829
cN0	24 (12.1%)	14 (13.3%)	10 (10.6%)	
cN1	105 (52.8%)	54 (51.4%)	51 (54.3%)	
cN2 or cN3	70 (35.2%)	37 (35.3%)	33 (35.1%)	
ER				0.026
Positive	117 (58.8%)	54 (51.4%)	63 (67.0%)	
Negative	82 (41.2%)	51 (48.6%)	31 (33.0%)	
PR				0.019
Positive	101 (50.8%)	45 (42.9%)	56 (59.6%)	
Negative	98 (49.2%)	60 (57.1%)	38 (40.4%)	
HER2				0.102
Positive	99 (49.7%)	58 (55.2%)	41 (43.6%)	
Negative	100 (50.3%)	47 (44.8%)	53 (56.4%)	
Molecular subtype				0.239
HR+/HER2–	66 (33.2%)	30 (28.6%)	36 (38.3%)	
HER2+	99 (49.7%)	58 (55.2%)	41 (43.6%)	
TN	34 (17.1%)	17 (16.2%)	17 (18.1%)	
Ki-67				0.048
<30	83 (41.7%)	37 (35.2%)	46 (48.9%)	
≥30	116 (58.3%)	68 (64.8%)	48 (51.1%)	

### Treatment to Patients

All patients completed 6–8 cycles of chemotherapy and underwent breast surgery based on the current National Comprehensive Cancer Network (NCCN) guideline (Goetz et al., 2019). All patients with HER2– breast cancer received four cycles of epirubicin and cyclophosphamide and then followed by four cycles of taxotere (EC-T regimen), whereas patients with HER2+ breast cancer were treated with four cycles of epirubicin and cyclophosphamide and followed by four cycles of taxotere and trastuzumab (EC-TH regimen), or received six cycles of taxotere, carboplatin, and trastuzumab (TCH regimen). For HER2+ breast cancer, some patients received trastuzumab and pertuzumab dual-target therapy during their NACT (EC-THP or TCbHP regimen). After NACT, BCS, or mammectomy were performed. The staging of axillary nodes included sentinel lymph node biopsy (SLNB) or axillary lymph node dissection (ALND).

### Pathological Assessment

All pre-NACT biopsy and postoperative pathological results were analyzed by a breast pathologist with more than 10 years of experience. The receptor status and Ki-67 index of the tumor were determined based on immunohistochemistry (IHC) staining. The HR was defined as positive for estrogen receptor (ER) or progesterone receptor (PR) expression when ≥1% of the tumor cells showed nuclear staining, and the HER2 expression graded 3+ was defined as positive while 0 and 1+ were negative (Hammond et al., 2010; Wolff et al., 2013). When the HER2 expression graded 2+ was reported, the gene amplification by the fluorescence *in situ* hybridization (FISH) was used to determine the HER2 status. Tumors were classified into three molecular subtypes as follows: (1) HR+/HER2–, (2) HER2+, and (3) TN. For Ki-67 index, we defined tumor cells with ≥30% staining as high expression and those with <30% staining as low expression.

**Figure 1:**
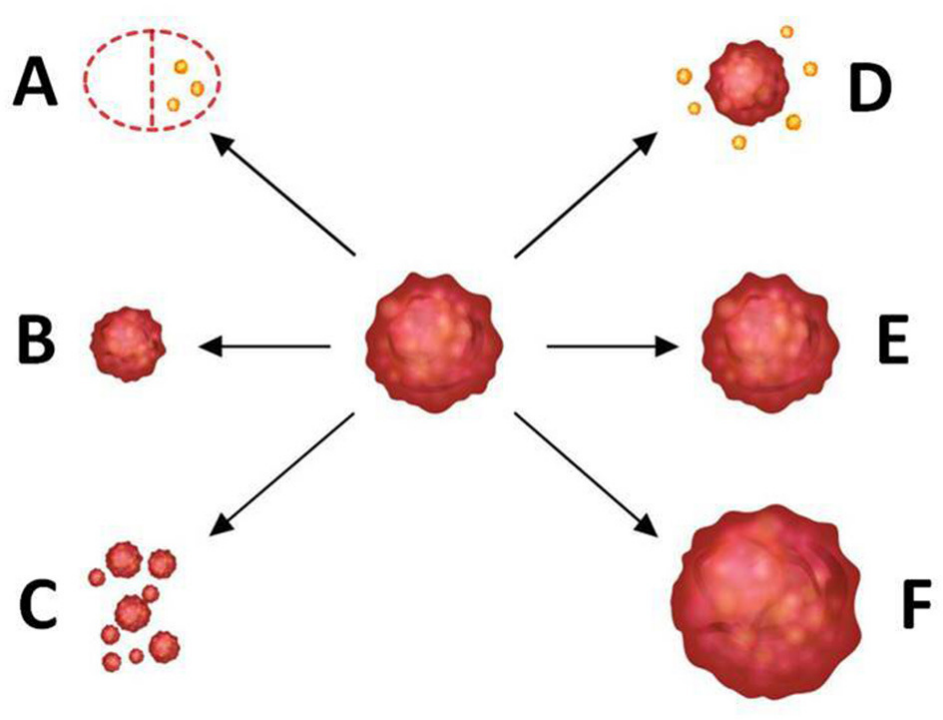
Tumor shrinkage patterns after neoadjuvant chemotherapy. **(A)** Pathological complete response [i.e., no residual tumor or only residual ductal carcinoma *in situ* (DCIS), defined as ypT_0/is_], **(B)** concentric shrinkage (i.e., only one residual invasive tumor focus, without DCIS), **(C)** multifocal shrinkage (i.e., more than 2 invasive tumor foci, with/without DCIS), **(D)** diffuse shrinkage (i.e., a main residual invasive focus with surrounding satellite DCIS), **(E)** stable disease (SD), and **(F)** progressive disease (PD). **(A,B)** belong to type 1 shrinkage pattern. **(C**–**F)** belong to type 2 shrinkage pattern.

The specimen pathology was used as the gold standard of tumor shrinkage pattern, and the largest diameter of the invasive tumor region on slides was measured by two pathologists in consensus. According to the surgical pathology, tumor shrinkage patterns were classified as follows: (a) pCR (i.e., no residual tumor or only residual ductal carcinoma in situs, defined as ypT_0/is_), (b) concentric shrinkage (i.e., only one residual invasive tumor focus, without DCIS), (c) diffuse shrinkage (i.e., a main residual invasive focus with surrounding satellite DCIS), (d) multifocal shrinkage (≥ 2 invasive tumor foci, with/without DCIS), (e) SD, and (f) PD. The former two are classified into type 1 shrinkage, which is called favorable shrinkage pattern, and the last four are classified into type 2 shrinkage, which is called the poor shrinkage pattern. Figure 1 shows the tumor shrinkage patterns after NACT.

The longest diameter of the primary tumor on the segmented 3D regions of interest (ROI) was measured in the MRI workstation as well. According to the Response Evaluation Criteria in Solid Tumors (RECIST 1.1) guideline, patients were classified into response or non-response group to NACT as the following: patients who responded to NACT were determined when the invasive tumor area showed a decrease of largest diameter ≥30% compared with that in the MRI, while SD indicated a decrease of largest diameter <30% or an increase of largest diameter <20%, and PD indicated an increase of largest diameter ≥20% (Chalian et al., 2011; Schwartz et al., 2016). Concentric shrinkage is defined as only one invasive tumor focus without DCIS, and such a shrinkage pattern is more likely to achieve the negative surgical margins in BCS, while multifocal and diffuse shrinkages are both considered as significant responses to NACT but still unsuitable for BCS.

### MRI Acquisition

All patients underwent MRI examination using a 3.0 Tesla system (Siemens Verio, syngo MR B17, Erlangen, Germany) with a dedicated 16-channel breast coil within 2 weeks prior to the initiation of NACT. The MRI sequences of each patient included as follows: an axial fat-suppressed T2-weighted imaging (T2WI), an axial T1-weighted DCE (T1-DCE), and an apparent diffusion coefficient (ADC) map derived from diffusion-weighted imaging. The details of the MRI examination and parameters for MRI images are shown in the Supplementary Material.

### Tumor Segmentation and Features Extraction

Two radiologists with more than 10 years of experience in breast imaging performed the tumor segmentation with Segmentation Module in 3D Slicer software (version 4.10.2, www.slicer.org) (Fedorov et al., 2012; Cheng et al., 2016). On T1-DCE images, the high signal intensity of the tumor region after injection of the contrast agent allows an accurate delineation to the tumor margins. The semi-automatic algorithms did the preliminary segmentation according to the intensity threshold segmentation, and then, manual corrections such as relabeling and hole-filling were done by two professional radiologists in consensus. The masks of ROI on T1-DCE were then registered to the other two MRI sequences (i.e., T2WI and ADC map). Finally, we got three segmentation ROI masks per patient. More details about the tumor segmentation are shown in the Supplementary Material.

Feature extraction of ROI was performed with Pyradiomics Module in 3D Slicer software (https://github.com/Radiomics/pyradiomics) (van Griethuysen et al., 2017; Zwanenburg et al., 2020). Before features extraction, the voxel size of each sequence was resampled to 1 ×1 ×1 mm, and the bin width of the gray-level histogram was fixed as 25. Six Laplacian of Gaussian filters (i.e., kernel sizes were set as 1, 2, 3, 4, 5, and 6) and a wavelet-based filter were used to process the original MRI images. Then, 1,424 quantitative radiomics features could be extracted from each MRI sequence, and the features were divided into seven categories: (1) first-order statistics features, (2) shape-based features, (3) gray-level co-occurrence matrix (GLCM), (4) gray-level size zone matrix (GLSZM), (5) gray-level run length matrix (GLRLM), (6) neighboring gray-tone difference matrix (NGTDM), and (7) gray-level dependence matrix (GLDM). After removing the duplicate shape of features, a total of 4,198 radiomics features from three MRI sequences could be extracted per patient. The information of the various features is shown in Supplementary Material. The clinical characteristics such as age, menopausal status, histological type, clinical anatomical TNM stage, ER status, PR status, HER2 status, and Ki-67 index were also added in the feature set.

### Feature Selection

Features selection was performed with Python 3.70 (https://www.python.org/). Before feature selection, the feature normalization was performed to ensure a relatively uniform range of all the radiomics features. The z-score normalization process is shown in the Supplementary Material. To achieve the dimensionality reduction, we used ANOVA and the least absolute shrinkage and selection operator (LASSO) logistic regression with 10-fold cross-validation to select the most significant features corresponding to the tumor shrinkage pattern. Then, the Pearson's correlation coefficient matrix (PCCM) was used to identify the multicollinearity between features. If there is any pair of features with a correlation coefficient of more than 0.85 or less than −0.85, then only one feature with a higher discriminative ability was selected. Finally, we selected the most significant features to make a combination with the best prediction performance.

### Establishment and Assessment of Models

The establishment and evaluation of the machine learning model were performed with Scikit-learn 0.18 package in Python 3.70. All patients were randomly allocated to the training dataset and testing dataset by stratified cross-validation, which included a 6-fold outer loop and a 5-fold inner loop. The positive/negative sample ratio was similar in the training dataset and testing dataset. In the outer loop, 5-fold (83.4%, 166 patients) dataset was used as the training dataset to develop the model, and the independent testing dataset (16.6%, 33 patients) was used to evaluate the model performance. To determine the best optimal hyperparameters, grid searching, and cross-validation were employed in the inner loop, and 1-fold (16.6%, 33 patients) dataset, also called the inner validation dataset, was assessed to choose the best hyperparameters. The whole process (i.e., stratified splitting, subsequent model development, hyperparameters optimization, and performance evaluation) was repeated by a 30-round bootstrap method to assess the robustness.

**Figure 2:**
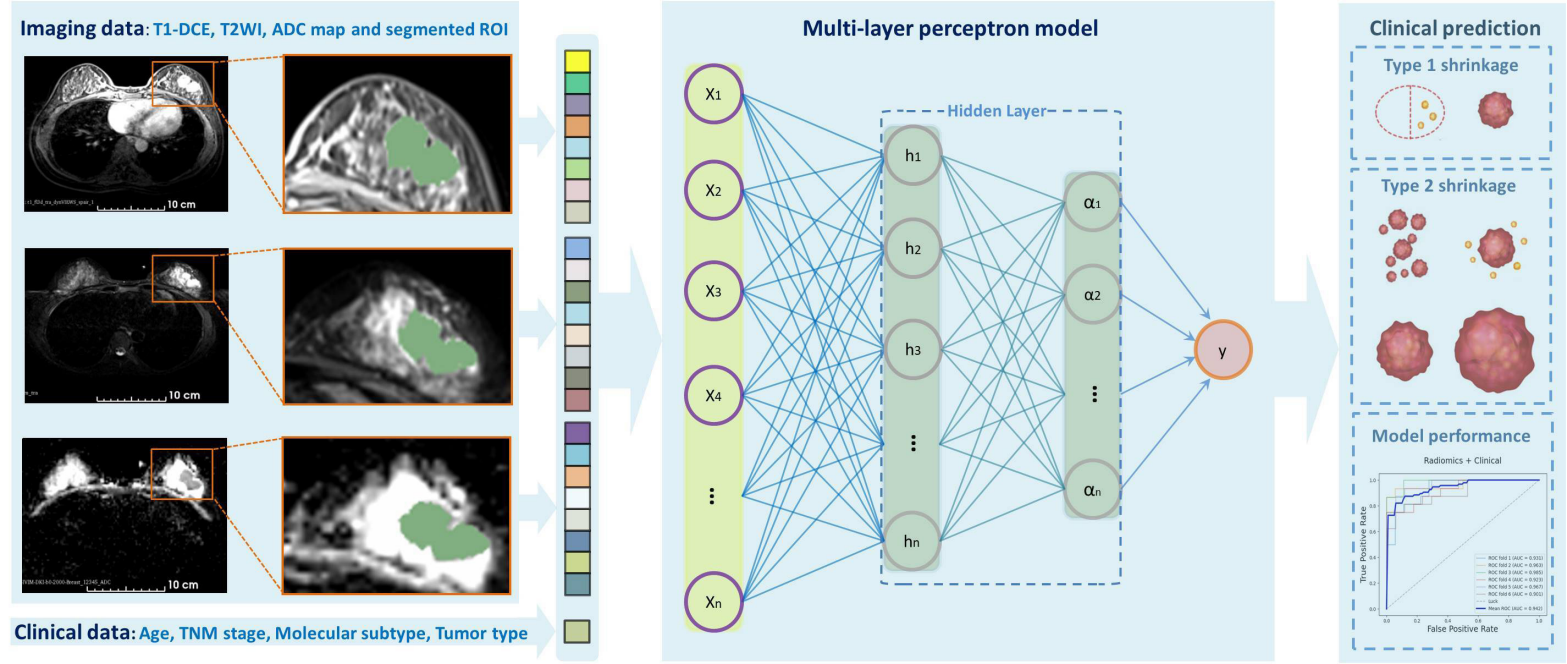
Outline of the workflow from the data input, feature extraction, selection, model construction, and performance assessment.

The clinical model and radiomics model were constructed with selected significant clinicopathologic and radiomics features, respectively, and then, the total features were combined to establish a combined model. As our model construction was a task for the labeled data, we used 12 robust supervised classification algorithms as following: Logistic Regression, Support Vector Machine (i.e., linear or radial kernel), Linear Discriminant Analysis, Random Forest, Extreme Gradient Boosting, Gaussian Naïve Bayes, AdaBoost, Decision Tree, K-Nearest Neighbors, AdaBoost, and Multilayer Perception (MLP) neural network. Then, the performance of each model was evaluated using the receiver operating characteristic (ROC) curves, the area under the ROC curve (AUC), accuracy, sensitivity, and specificity. Each specific algorithm was designed to fit the training dataset in the inner loop and to correctly predict the independent testing dataset in the outer loop. The prediction results were used to assess the performance and generalization ability of the models. We divided our patients into three subgroups according to molecular subtypes as follows: (1) HR+/HER2–, (2) HER2+, and (3) TN. The stratified 5-fold cross-validation was used to evaluate the model performance for each subtype. Figure 2 shows the workflow of data input, feature extraction, selection, model construction, and performance assessment.

### Statistical Analysis

The baseline data of the patient were evaluated with professional statistics packages in Python 3.7.0 and SPSS (version 20.0). The quantitative data were calculated and recorded as mean ± SD, and the qualitative data were summarized as frequencies and percentages. The Mann–Whitney *U*-test or Student's *t*-test was used for quantitative variables, and the chi-squared test or Fisher's exact test was used for qualitative variables. The normality test and *Z*-test were done for the comparison of performance indexes. The discrimination metrics of models, such as AUC, accuracy, sensitivity, and specificity, were also calculated. A two-sided *p* < 0.05 is considered statistically significant. The Wilson score interval method was used to calculate the CI of AUC.

## Results

### Baseline Characteristics of Patients

In total, 199 eligible patients were enrolled in this study. The baseline data are presented in Table 1. The mean age was 46.85 ± 10.13 years (range 23–78 years), and 116 patients (58.3%) were premenopausal women. Among all patients, 66 were HR+/HER2– (33.2%), 99 were HER2+ (49.7%), and 34 were TN (17.1%). After NACT, 105 patients had achieved type 1 tumor shrinkage pattern (52.8%), whereas 94 had achieved type 2 tumor shrinkage pattern (47.2%) according to the histological confirmation. Significant differences of some baseline characteristics were detected between two groups, including ER, PR, and Ki-67 (*p* = 0.026, 0.019, and 0.049, respectively).

### Feature Extraction and Selection

**Figure 3:**
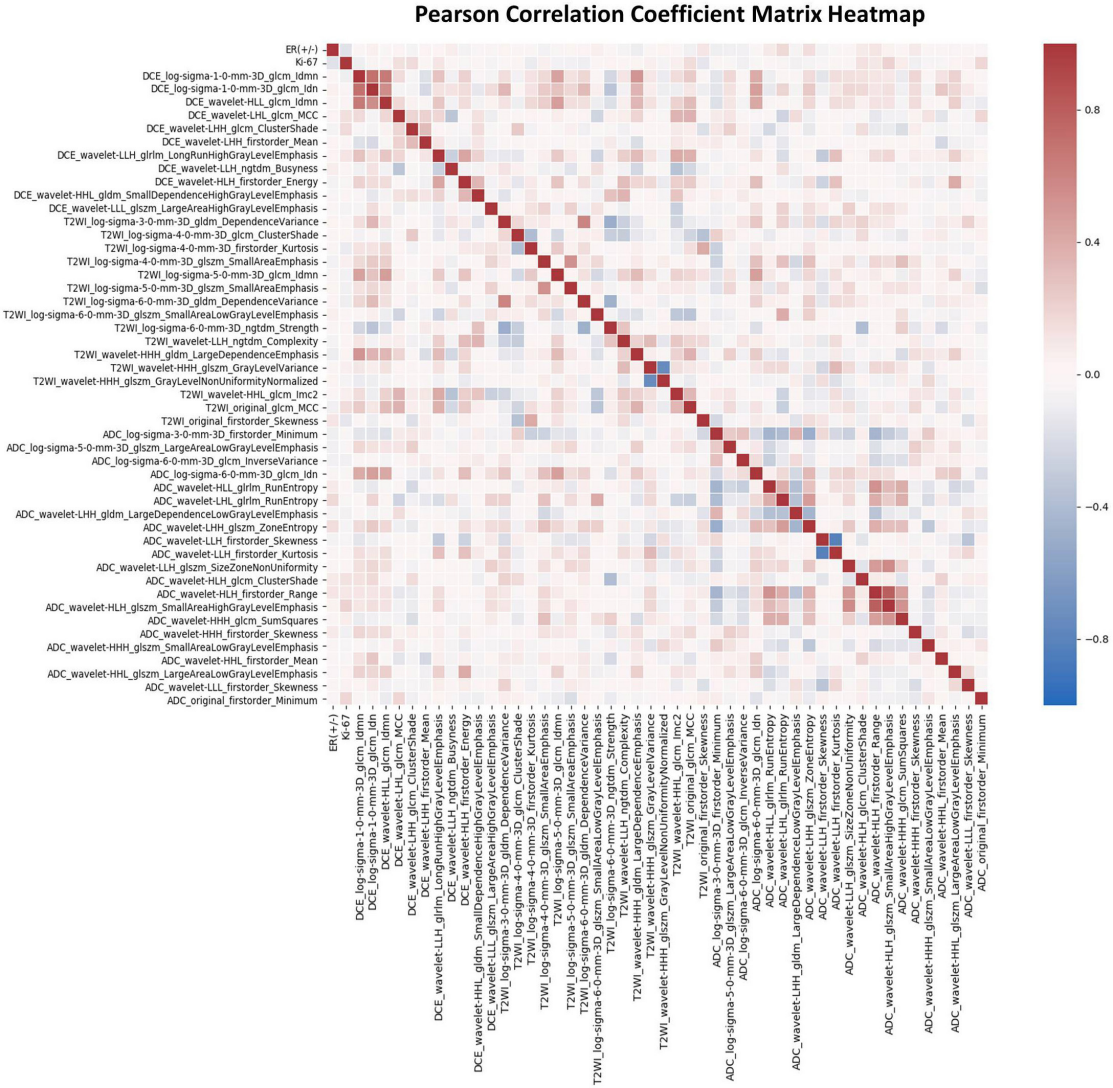
The Pearson's correlation coefficient matrix of the selected features. Red color denotes positive correlation, blue denotes a negative correlation, and the shade of the color indicates the correlation intensity.

For each patient, 4,198 radiomics features and 10 clinical features were used in the followed machine learning process. The ANOVA and LASSO logistic regression were used to reduce dependency and redundancy, and finally, the 50 most optimal features were selected as follows: 2 clinicopathologic characteristics (ER and Ki-67), and 48 radiomics features, including 11, 16, and 21 features from T1-DCE, T2WI, and ADC map, respectively. The associations of these features were assessed using the PCCM Heatmap, which is shown in Figure 3. The features were considered independent of each other as there was no PCC value over 0.85 or < −0.85.

### Development and Performance of Models

In order to find the most suitable algorithm for the prediction of tumor shrinkage patterns, 12 robust machine learning algorithms were applied based on the total features we selected. Table 2 summarizes the performances of each algorithm. The MLP neural network outperformed all other classifier algorithms, with a mean AUC value of 0.939 (95% CI: 0.896–0.965), a mean accuracy of 0.870 (95% CI: 0.815–0.910), a mean sensitivity of 0.840 (95% CI: 0.781–0.885), and a mean specificity of 0.897 (95% CI: 0.846–0.933) in the testing dataset based on the 30-round bootstrap validation. Then, we chose the MLP neural network as the basic algorithm to construct the machine learning models. The MLP neural network contained input, hidden, and output layers, and the hyperparameters in each layer were trained in the inner loop. The prediction workflow of MLP classifier was as follows: all features of one patient were input to the first layer, and finally, the output layer provided a prediction result.

**Table 2:** Performances of the 12 machine learning classifiers on 30-round 6-fold cross-validation in testing dataset for predicting tumor shrinkage pattern based on all the selected features.

**Algorithm**	**AUC (95% CI)**	**Accuracy (95% CI)**	**Sensitivity (95% CI)**	**Specificity (95% CI)**
MLP neural network	0.939 (0.896–0.965)	0.870 (0.815–0.910)	0.840 (0.781–0.885)	0.897 (0.846–0.932)
Logistic Regression	0.922 (0.874–0.952)	0.847 (0.789–0.891)	0.817 (0.756–0.865)	0.874 (0.819–0.913)
SVM (radial kernel)	0.909 (0.859–0.941)	0.823 (0.761–0.869)	0.784 (0.720–0.836)	0.855 (0.798–0.898)
Linear discriminant analysis	0.902 (0.851–0.936)	0.815 (0.753–0.863)	0.789 (0.725–0.840)	0.838 (0.779–0.883)
SVM (linear kernel)	0.890 (0.837–0.926)	0.809 (0.747–0.858)	0.780 (0.715–0.832)	0.835 (0.775–0.880)
Random forest	0.803 (0.740–0.852)	0.731 (0.663–0.788)	0.664 (0.594–0.727)	0.790 (0.726–0.841)
Gradient boosting	0.786 (0.722–0.838)	0.712 (0.644–0.771)	0.650 (0.580–0.714)	0.769 (0.703–0.822)
K neighbors	0.782 (0.718–0.834)	0.722 (0.654–0.780)	0.633 (0.562–0.698)	0.802 (0.739–0.852)
Gaussian NB	0.776 (0.711–0.829)	0.667 (0.597–0.730)	0.775 (0.710–0.828)	0.570 (0.499–0.638)
AdaBoost	0.769 (0.704–0.823)	0.708 (0.640–0.768)	0.679 (0.610–0.741)	0.734 (0.667–0.791)
XGB	0.758 (0.692–0.813)	0.677 (0.607–0.739)	0.639 (0.568–0.703)	0.711 (0.643–0.770)
Decision tree	0.630 (0.559–0.695)	0.630 (0.559–0.694)	0.630 (0.559–0.695)	0.629 (0.559–0.694)

Based on the MLP neural network, nine various models based on feature type were constructed and the prediction performances of various models were shown in Table 3. The accuracy of Model_Radiomics_ was 0.828 (95% CI: 0.767–0.874), which demonstrated a better performance than that in Model_T1−DCE_ (0.644, 95% CI: 0.573–0.708), Model_T2WI_ (0.606, 95% CI: 0.534–0.672), Model_ADCmap_ (0.709, 95% CI: 0.641–0.768), and Model_Clinical_ (0.561, 95% CI: 0.490–0.629). The AUC value (0.900, 95% CI: 0.849–0.935) of Model_Radiomics_ also outperformed that in the other four models (i.e., Model_T1−DCE_: 0.712, 95% CI: 0.644–0.771; Model_T2WI_: 0.661, 95% CI: 0.591–0.724; Model_ADCmap_: 0.795, 95% CI: 0.732–0.846; and Model_Clinical_: 0.611, 95% CI: 0.540–0.677) on testing dataset. On the training dataset, the Model_Radiomics_ achieved a mean accuracy of 0.912 and a mean AUC of 0.975. When the clinicopathologic characteristics were added to construct Model_Radiomics+Clinical_, the accuracy was improved to 0.870 (95% CI: 0.815–0.911) and the AUC was improved to 0.939 (95% CI: 0.896–0.965) on testing dataset, which showed the highest performance in differentiating tumor shrinkage pattern. On the training dataset, the Model_Radiomics+Clinical_ achieved a mean accuracy of 0.930 and a mean AUC of 0.985.

**Table 3:** Diagnostic performances to classify tumor shrinkage pattern in testing dataset of different models based on the type of features using the Multilayer Perception (MLP) neural network.

**Model**	**AUC (95%CI)**	**Accuracy (95%CI)**	**Sensitivity (95%CI)**	**Specificity (95%CI)**
Model_**T1−DCE**_	0.712 (0.644–0.771)	0.644 (0.573–0.708)	0.489 (0.418–0.558)	0.783 (0.719–0.835)
Model_**T2WI**_	0.661 (0.591–0.724)	0.606 (0.534–0.672)	0.562 (0.491–0.630)	0.645 (0.575–0.709)
Model_**ADCmap**_	0.795 (0.732–0.846)	0.709 (0.641–0.768)	0.699 (0.630–0.759)	0.718 (0.650–0.777)
Model_**Clinical**_	0.611 (0.540–0.677)	0.561 (0.489–0.629)	0.653 (0.582–0.716)	0.480 (0.410–0.550)
Model_**Radiomics**_	0.900 (0.849–0.935)	0.828 (0.767–0.874)	0.788 (0.724–0.839)	0.864 (0.807–0.905)
Model_**T1−DCE+Clinical**_	0.743 (0.676–0.799)	0.687 (0.618–0.748)	0.713 (0.644–0.771)	0.665 (0.595–0.727)
Model_**T2WI+Clinical**_	0.708 (0.640–0.768)	0.649 (0.579–0.713)	0.627 (0.556–0.692)	0.670 (0.599–0.732)
Model_**ADCmap+Clinical**_	0.809 (0.746–0.858)	0.729 (0.662–0.787)	0.699 (0.630–0.759)	0.757 (0.690–0.811)
Model_**Radiomics+Clinical**_	0.939 (0.896–0.965)	0.870 (0.815–0.910)	0.840 (0.781–0.885)	0.897 (0.846–0.932)

**Figure 4:**
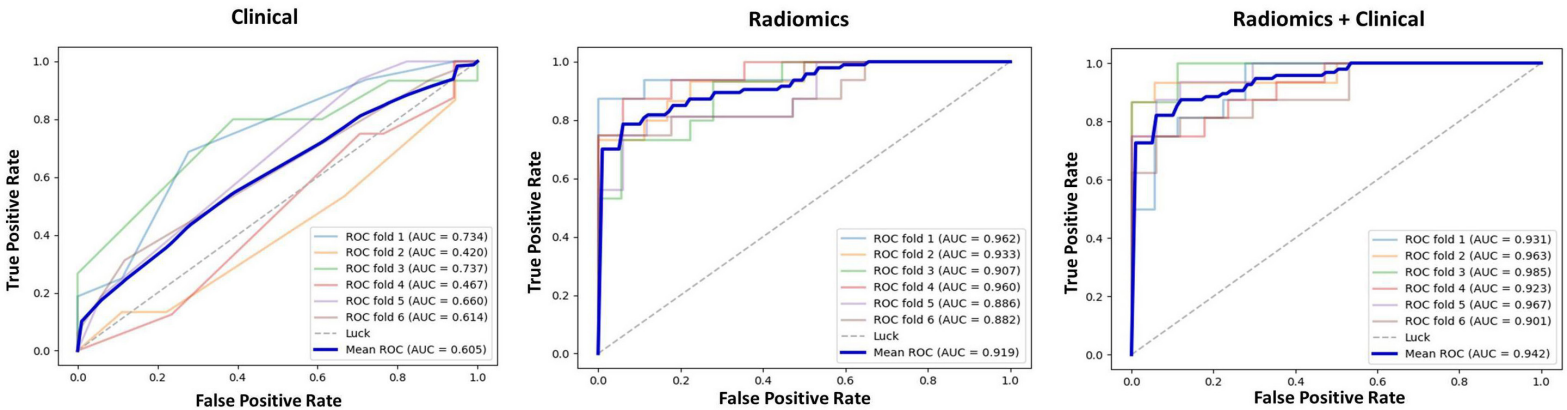
Receiver operating characteristic (ROC) curves of clinical prediction model, radiomics prediction model, and combined prediction model in testing dataset for 1-round cross-validation.

**Figure 5:**
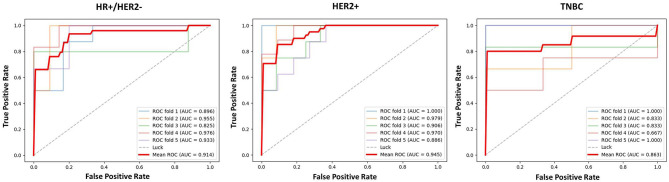
The ROC curves of prediction model based on three molecular subtypes in testing dataset for 1-round cross-validation.

The results of 1-round 6-fold cross-validation of ROC curves of three representative models (i.e., Model_Clinical_, Model_Radiomics_, and Model_Radiomics+Clinical_) are shown in Figure 4. For the different molecular subtypes, the predictive performance was evaluated, respectively, and the results were shown in Table 4. The HER2+ subtype achieved the highest performance in Model_Radiomics+Clinical_ with an AUC value of 0.940 (95% CI: 0.873–0.973), while the TN subtype had a relatively low performance with an AUC value of 0.837 (95% CI: 0.699-1.0) in the testing dataset. The results of 1-round 5-fold cross-validation of ROC curves for three molecular subtypes are shown in Figure 5.

**Table 4:** Performance to classify tumor shrinkage pattern in three molecular subtypes.

**Molecular subtype**	**Training dataset**				**Testing dataset**			
	**AUC ** ** (95%CI)**	**Accuracy ** ** (95%CI)**	**Sensitivity ** ** (95%CI)**	**Specificity ** ** (95%CI)**	**AUC ** **(95%CI)**	**Accuracy ** **(95%CI)**	**Sensitivity ** **(95%CI)**	**Specificity ** **(95%CI)**
HR+/HER2-	0.999 (0.980–0.999)	0.991 (0.899–0.995)	0.999 (0.850–0.991)	0.986 (0.930–1.0)	0.901 (0.866–0.936)	0.816 (0.715–0.917)	0.729 (0.546–0.912)	0.883 (0.799–0.967)
HER+	0.999 (0.986–0.997)	0.987 (0.926–0.996)	0.997 (0.956–1.0)	0.980 (0.886–0.994)	0.940 (0.886–0.994)	0.865 (0.761–0.986)	0.912 (0.701–0.994)	0.799 (0.707–0.891)
TN	1.0 (0.913–1.0)	0.999 (0.767–0.967)	0.999 (0.688–1.0)	1.0 (0.773–1.0)	0.837 (0.699–0.975)	0.811 (0.614–0.993)	0.777 (0.456–0.993)	0.851 (0.558–0.996)

### The Stability and Interpretability of Models

**Figure 6:**
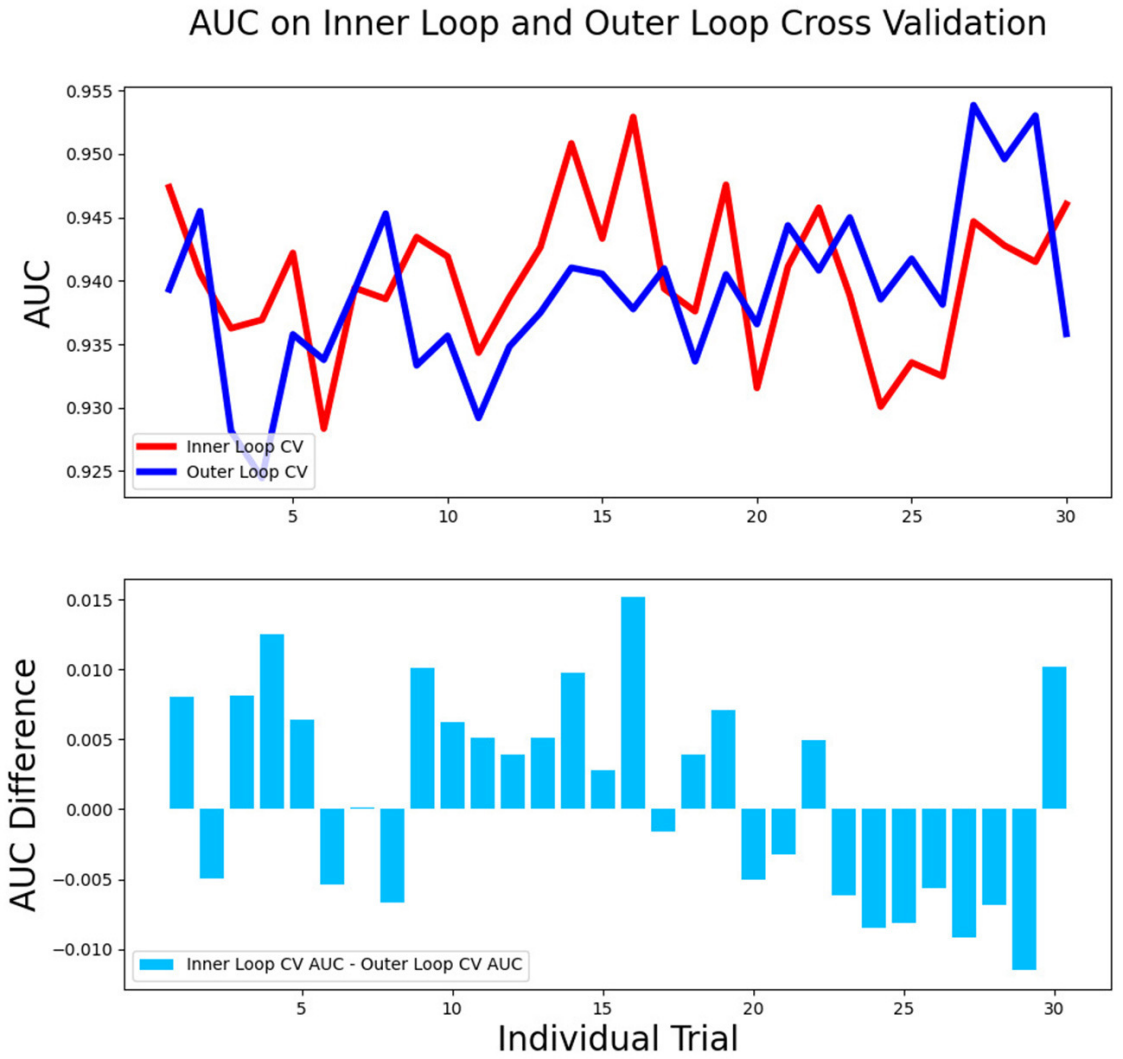
The AUC between the validation dataset in the inner loop and testing dataset in the outer loop for 30-round cross-validation.

**Figure 7:**
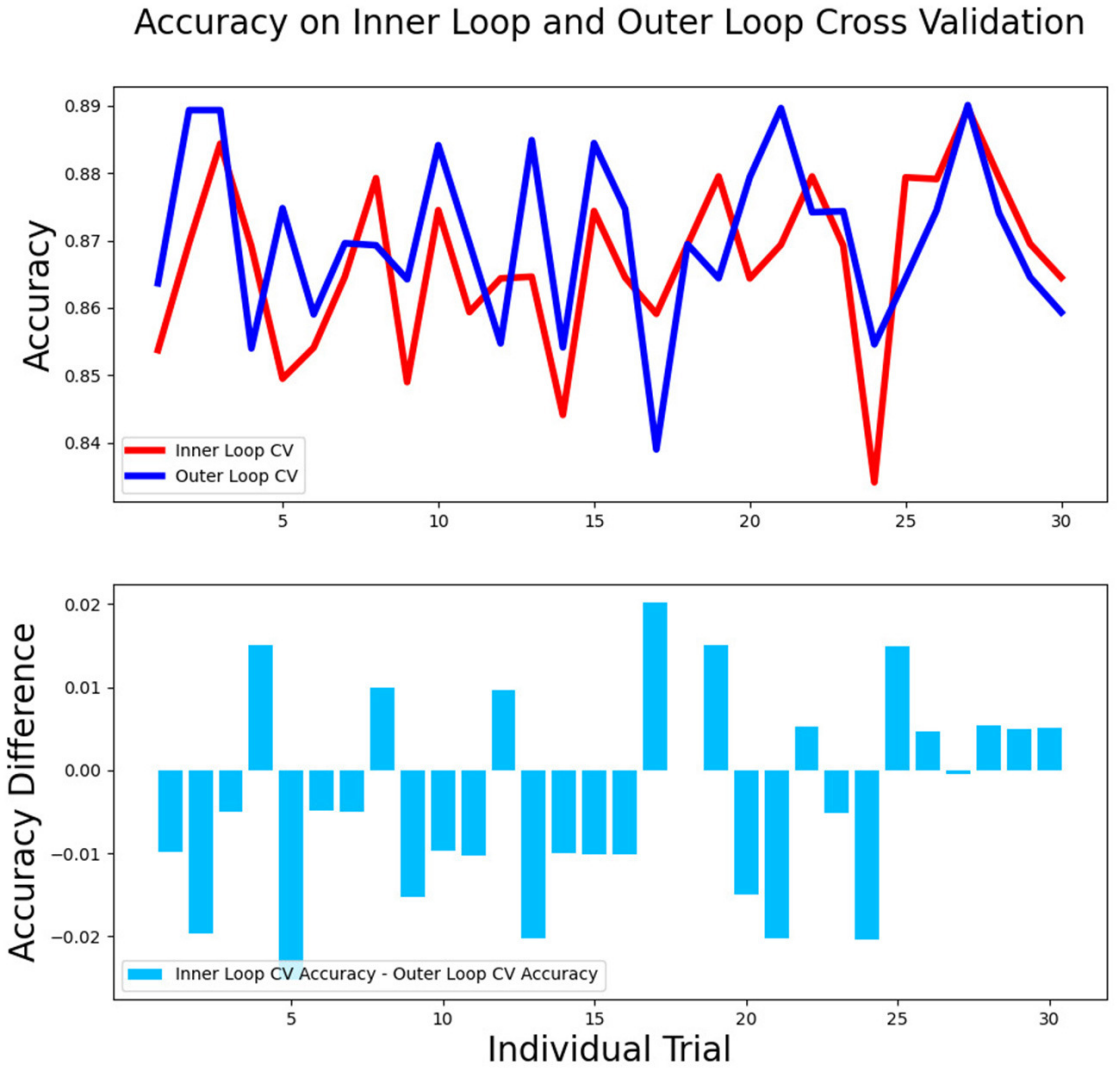
The accuracy between the validation dataset in the inner loop and testing dataset in the outer loop for 30-round cross-validation.

**Figure 8:**
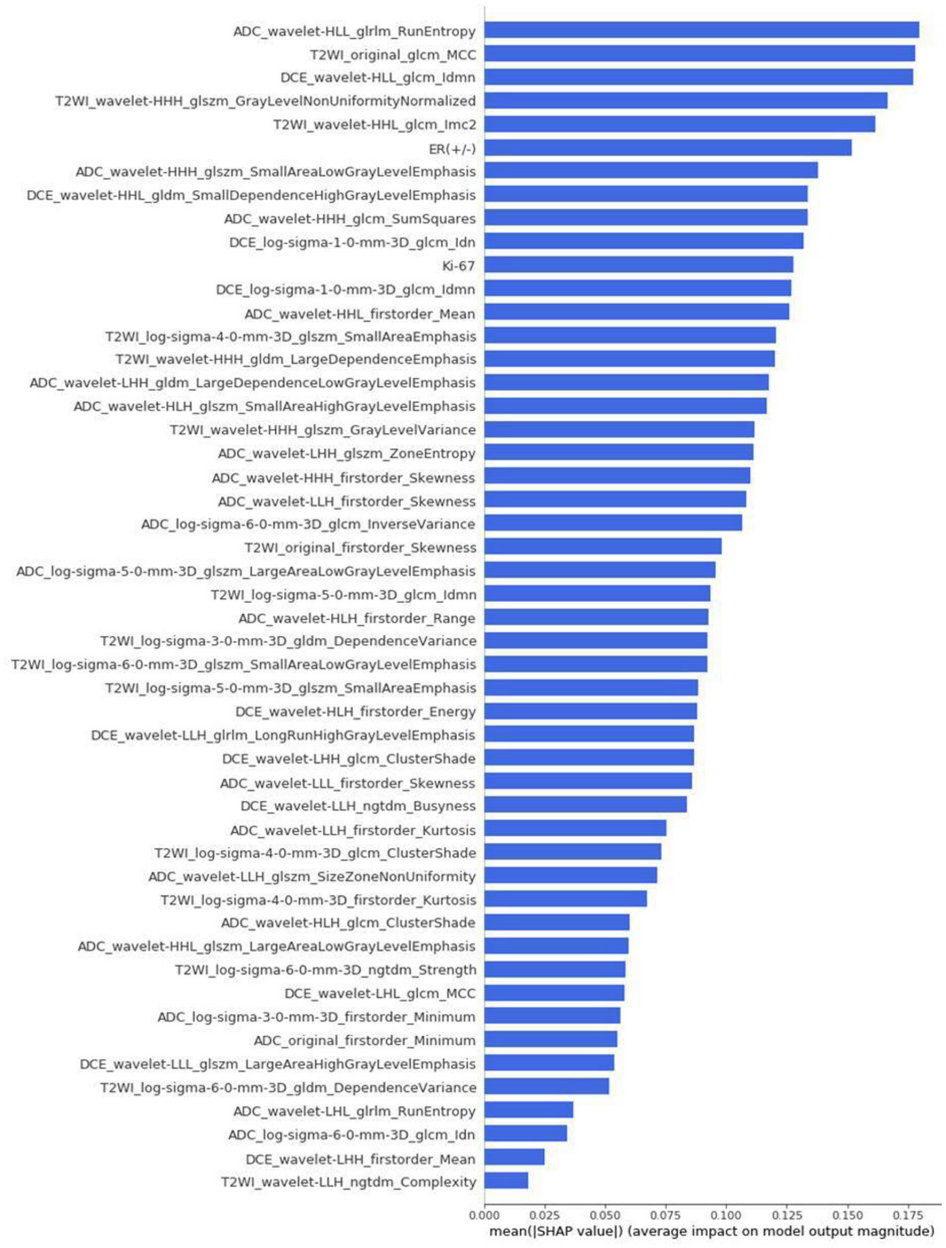
The weight importance rank of selected features. The feature with a longer bar contributes more to the model.

**Figure 9:**
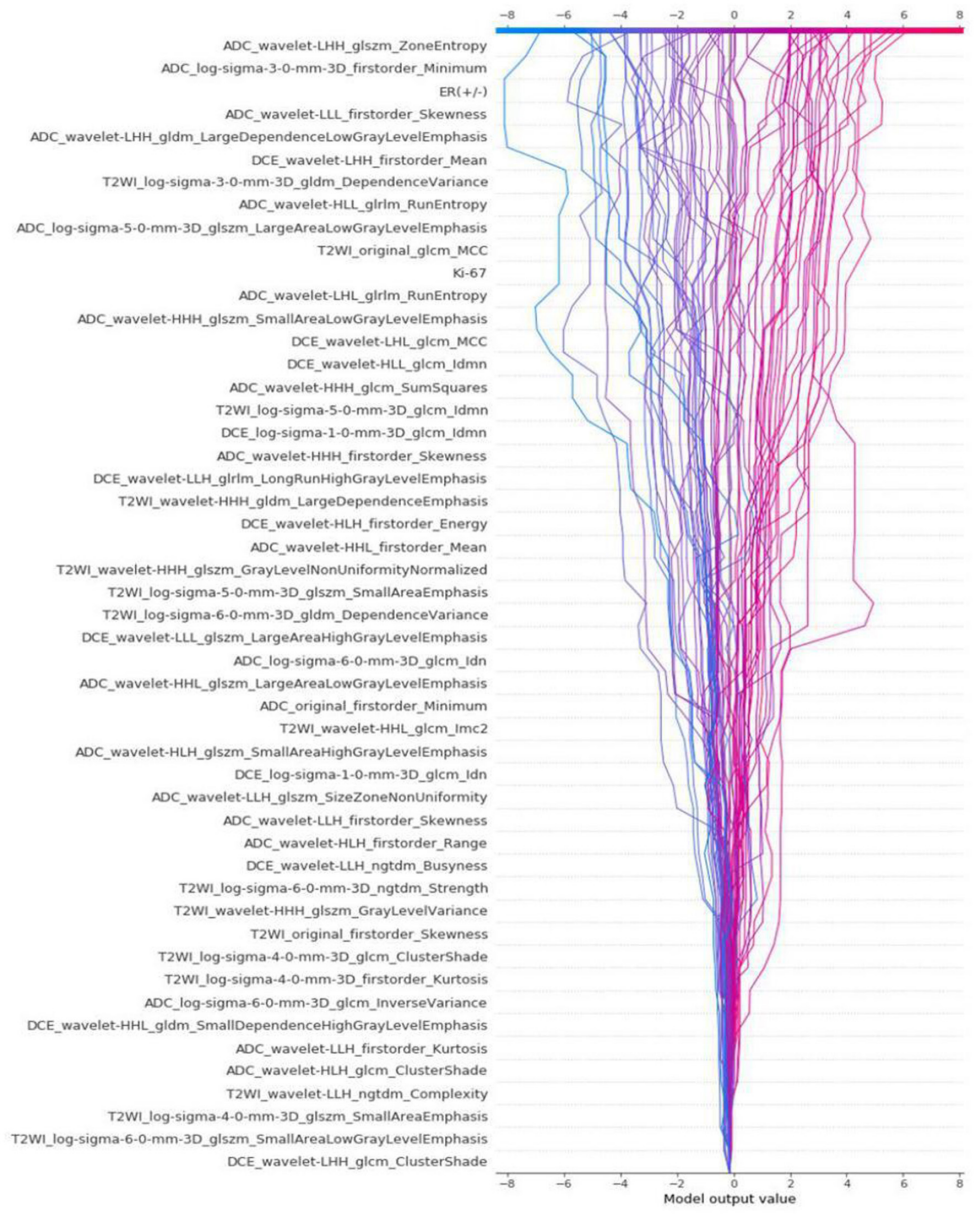
The decision curve that reflects how each feature affects the predictive output for the patients in testing dataset by classical splitting with a ratio of 7:3.

The differences in accuracy and AUC between the validation dataset in the inner loop and the testing dataset in the outer loop were calculated 30 times to assess the reproducibility of the results. There are no significant difference in AUC and accuracy between the outer loop and inner loop (Figures 6, 7), so we considered that the results of our models are stabilized and representative. To evaluate the feature importance and predictive workflow of our model, the SHapley Additive exPlanations (SHAP) values were calculated (Rodríguez-Pérez and Bajorath, 2020a,b). The mean SHAP value of each feature was summarized based on its weight importance to the model, which calculated the number of times a feature was used to split the dataset in the model. For the prediction of tumor shrinkage pattern, each selected feature had a significant impact on the model output. Figure 8 lists the weight importance rank of the total features. Figure 9 shows the decision curve reflecting how each feature affects the predictive output for the patients in the testing dataset (i.e., one round of classical splitting method with a ratio of 7:3). The baseline SHAP value was set as 0, and in the workflow of our model, each feature has a positive or negative impact on the final output value. When the output value was over 0, the patient was considered achieving type 1 shrinkage pattern after NACT, and when the value was <0, type 2 shrinkage pattern was more likely considered.

## Discussion

As there is an increasing need for BCS in patients with breast cancer, the accurate evaluation of tumor shrinkage patterns prior to NACT in a non-invasive way is essential. The aim of this study was to investigate the relationship among tumor shrinkage patterns, clinicopathologic characteristics, and MRI-derived radiomics features. We aimed to develop a model to assess tumor shrinkage patterns prior to NACT. The radiomics method could improve the diagnostic accuracy of MRI for tumor response to NACT. Enabling the prediction of tumor shrinkage pattern prior to treatment would help determine the feasibility of BCS and may lead to alterations in chemotherapy regimen or performing surgery earlier than initially planned.

The NACT benefits those patients who are willing to have BCS but the tumor size is large and not suitable for the surgery (Hennessy et al., 2005; Mathew et al., 2009; Mougalian et al., 2016). Wolmark et al. have reported that the ipsilateral recurrence rate of patients treated with BCS after NACT was 10.7%, and the rates were 7.6% in patients with primary tumors fit for BCS and 15.9% in patients with primary breast cancer unfit for BCS (Wolmark et al., 2001). Tumor downstaged by NACT and followed by BCS has a higher local recurrence rate than the primary tumor and is fit for BCS, which may be a result of incomplete resection of cancer cells. It has been clearly indicated that a clear surgical margin is essential to decrease the local recurrence rate. In clinical practice, however, the current criteria for the evaluation of tumor response, the RECIST 1.1, are used extensively to assess tumor response to NACT, but it cannot identify those patients with tumor concentric shrinkage (Chalian et al., 2011; Schwartz et al., 2016).

The MRI examination can accurately show the morphology and extent of breast cancer, and it guides surgical decisions to ensure a negative surgical margin. Several studies have divided tumor shrinkage into concentric shrinkage and dendritic shrinkage based on the MRI examination (Wang et al., 2013; Ballesio et al., 2017; Fukada et al., 2018; Goorts et al., 2018; Zhang et al., 2018; Zhuang et al., 2020). According to earlier studies, after NACT, about 70% of residual tumors were concentric and 30% were dendritic in MRI images. Post-NACT tumors that showed pCR and concentric shrinkage were easier to obtain negative surgical margins, so those patients were the candidates of BCS (Chen et al., 2004). In general, the tumor that shows dendritic shrinkage has multicentric and discontinuous residual tumor, which can cause postoperative local recurrence and metastasis, so it is unfit for BCS. However, some solitary residual tumors may be missed by conventional histological sections, and negative margins are still observed in surgical specimens. Favorable tumor shrinkage patterns, such as pCR and concentric shrinkage tumor after NACT, were included in the standards for BCS, while patients with poor shrinkage pattern show either multifocal residual tumors or no significant decline in tumor size. The post-NACT pCR is more difficult to obtain in luminal breast cancer than other subtypes, and dendritic shrinkage and mixed shrinkage are also more common in the luminal subtype. The accurate assessment of tumor shrinkage pattern can help in choosing an optimal treatment option for patients. To find patients suitable for BCS, our study divided the tumor shrinkage patterns into two types as well. Patients with type 1 shrinkage pattern showed an adequate response to NACT, with tumor complete remission or a significant decline in size. In our study, the percentages of type 1 shrinkage pattern in three subtypes were as follows: HR+/HER2– (45.5%), HER2+ (58.6%), and TN (50%). Consistent with the earlier studies, the concentric shrinkage patterns were more likely to occur in patients with HER2+ and TN subtype tumors.

The assessment of tumor size using MRI during NACT is a good predictor of the tumor response to NACT. Loo et al. reported that MRI was useful to monitor tumor response during NACT and massive tumor regression was more easily observed in HER2+ and TN tumors than in HR+/HER2– tumors (Loo et al., 2011). In a meta-analysis of Yuan et al., MRI had high specificity (91%) and relatively low sensitivity (63%) in predicting pCR after NACT in patients with breast cancer (Yuan et al., 2010). Liu et al. had reported that a radiomics model combining T1-DCE, DWI, and T2WI images had a great performance to predict the tumor response to NACT and achieved an AUC of 0.71–0.80 in the testing cohort, but the literature did not identify those patients with concentric shrinkage after NACT (Liu et al., 2019). Zhuang et al. established a nomogram to predict the tumor regression pattern using T2WI, DWI sequences, and clinical factors, and their model achieved an AUC of 0.826 in the testing cohort (Zhuang et al., 2020). Some studies have shown that the radiomics models had diagnostic value in tumor response to NACT, and most of the literature aimed to distinguish pCR and non-pCR (Antunovic et al., 2019; Liu et al., 2019; Li et al., 2020; Zhuang et al., 2020). Fukada et al. reported that the shrinkage pattern at MRI during NACT was the significant independent predictor and the radiomics features based on MRI had closer associations with the risk of recurrence and prognosis in low-grade early-stage luminal breast cancer (Fukada et al., 2018). Assessing tumor response to NACT has been reported, which could predict the prognosis of patients with luminal breast cancer. Richard et al. found that the patients with breast cancer with a high pretreatment ADC in DWI were more likely to respond completely to NACT (Richard et al., 2013). From the earlier studies in the literature, we knew that mpMRI had the potential in assessing tumor shrinkage pattern, so in our study, we extracted radiomics features from three MRI sequences (i.e., T1-DCE, T2WI, and ADC map), and we also added the clinicopathologic characteristics into the feature set.

The radiomics method offers the great potential to identify the tumor shrinkage pattern prior to NACT, whereas the clinical characteristics provide limited information about the tumor. There continues to face a challenge for the success of BCS, and there is still a lack of effective methods to assess the tumor response during NACT and risk of local recurrence postoperatively. In our study, after the feature selection, 50 features, including the clinicopathologic characteristics and the radiomics features from MRI were selected to develop the machine learning model. The mean AUC of the Model_Clinical_ in the testing set was 0.611 (95% CI: 0.540–0.677), while the result of Model_Radiomics_ was 0.900 (95% CI: 0.849–0.935), and when combining the clinicopathologic characteristics with radiomics features, the result could rise to 0.939 (95% CI of the Model_Radiomics+Clinical_: 0.896–0.965). We also found that when combining the clinical characteristics with radiomics features, the model had a more stable performance with a lower SD. Our radiomics model that combined the clinical and radiomics features might provide a more accurate assessment for tumor shrinkage pattern prior to NACT treatment and is worthy of further study.

This study has some limitations. First, our study was based on a retrospective design and the patient population is limited, and it is better if there are data from external institutions that could validate our model. Actually, NACT was mainly used in locally advanced breast cancer, and it spent several months of a patient to complete the standard NACT and followed surgery, so the patient population is limited in most studies. Second, the distribution of molecular subtypes was imbalanced due to less number of patients with TN breast cancer. But in the total population with breast cancer, the TN subtype occupied the lowest proportion, which could explain the imbalanced distribution of our patients. Third, only the pre-NACT MRI data were collected to construct models, and it is worthwhile to study further of the predictive potential to the tumor shrinkage patterns based on the sequential MRI examination during NACT. Delta-radiomics that combines pre-NACT with the early-NACT MRI data could provide tumor response information in the early stage of treatment, and that combines pre-NACT with the post-NACT MRI data could help distinguish pCR from radial complete response.

## Conclusions

We constructed a model combining clinicopathologic characteristics and radiomics features to accurately predict tumor shrinkage pattern prior to NACT using the mpMRI data. The model performed well in different molecular subtypes, and this early prediction model can help clinicians make a clinical decision with the potential to evaluate the feasibility of BCS after effective chemotherapy. Further multicenter study with larger datasets could improve our prediction model and explore the potential for clinical application to the wider regions and population.

## Data Availability Statement

The datasets presented in this article are not readily available because of the privacy of patients and the huge amount of MRI data. Requests to access the datasets should be directed to linying3@mail.sysu.edu.cn.

## Ethics Statement

The studies involving human participants were reviewed and approved by the Institutional Ethics Review Board of the first affiliated hospital of Sun Yat-sen University. The patients/participants provided their written informed consent to participate in this study.

## Author Contributions

YH contributed to concept development, study management, data curation, literature searching, and writing the original draft. WC contributed to the study management, literature searching, methodology, software using, and language editing. XZ contributed to the analysis of breast imaging and writing the original draft. SH contributed to the data curation, imaging processing, and methodology. NS contributed to the concept development, study management, and writing the original draft. HS contributed to the analysis of pathology. ZL contributed to the statistical analysis and writing statistics section of the manuscript. XW and TL contributed to the data curation. HL and YL contributed to the concept development, funding acquisition, and writing the original draft. All authors contributed to the critical review and approved the submitted version of the article.

## Conflict of Interest

The authors declare that the research was conducted in the absence of any commercial or financial relationships that could be construed as a potential conflict of interest.

## Abbreviations

NACT: neoadjuvant chemotherapy
BCS: breast-conserving surgery
DCIS: ductal carcinoma *in situ*
IHC: immunohistochemistry
FISH: fluorescence *in situ* hybridization
mpMRI: multiparametric MRI
T1-DCE: T1-weighted dynamic contrast-enhanced imaging
T2WI: T2-weighted imaging
ADC: apparent diffusion coefficient
ROI: regions of interest
LASSO: least absolute shrinkage and selection operator
MLP: Multilayer Perception
HER2: human epidermal growth factor receptor 2
TN: triple-negative
HR: hormone receptor
ROC: receiver operating characteristic
AUC: area under the ROC curve
SLNB: sentinel lymph node biopsy
ALND: axillary lymph node dissection
pCR: pathological complete response
SD: stable disease
PD: progressive disease
PCCM: Pearson's correlation coefficient matrix.
