# Supplementary material for: Prediction of Tumor Shrinkage Pattern to Neoadjuvant Chemotherapy Using a Multiparametric MRI-Based Machine Learning Model in Patients With Breast Cancer
Source: Front Bioeng Biotechnol. 2021 Jul 6;9:662749. doi: 10.3389/fbioe.2021.662749 (PMC8291046; doi:10.3389/fbioe.2021.662749)
Supplement: Supplementary file 1 [file Data_Sheet_1.pdf]

Supporting Material I: MRI Examination and parameters:

The patients which we retrospectively recruited all underwent MRI scan a 3.0 Tesla system (Siemens Verio, syngo MR B17, Erlangen, Germany) with a dedicated 16-channel breast coil. The image acquisition protocol included fat-suppressed T2WI, fat-suppressed T1+DCE and ADC map images. Following was the detailed description of the data acquisition procedure. At the scanning, an axial fat-suppressed T2WI sequence and axial DWI images were obtained using four b values (0, 800, 1000 and 2000 s/mm2) were acquired before contrast medium administration. An initial fat-saturated T2WI and T1WI pre-contrast scan was collected before T1+DCE images scanning, and then T1+DCE images were acquired as six post contrast scans at intervals of 90 seconds following the intravenous injection of gadolinium contrast agent. A gadolinium-based agent (Magnevist; Bayer Healthcare, Berlin, Germany) was injected using an MR imaging compatible power injector at a rate of 2 ml/s and at a dose of 0.2 ml/kg of body weight, followed by a 20-ml saline flush with high-pressure injector. ADC map was derived from DWI imaging by two b values (the first b value was 0, and the second b value was 800, 1000 or 2000). Because the second b value was ⩾ 800, then we considered the ADC map was not influenced by the b values, and it can reflect the biological information of tumors in imaging accurately.

The parameters for each MRI image were as follows: (1) T2WI: repetition time/echo time = 4330/61 msec, pixel bandwidth = 319, FOV = 380×380 mm^2, Matrix = 320 × 320, flip angle = 80°, spatial resolution = 1.188 × 1.188 mm, slice thickness = 3.0 mm, slices: 38, axial orientation. (2) T1-DCE: repetition time/echo time = 4.32/1.57 msec, pixel bandwidth = 446, FOV = 380 × 380 mm^2, matrix = 448 × 448, flip angle = 10°, spatial resolution = 0.848 × 0.848 mm, slice thickness = 1.0 mm, slices: 144, axial orientation. (3) ADC map: repetition time/echo time = 6300/74 msec, pixel bandwidth = 2083, FOV = 380×380 mm^2, Matrix = 160 × 160, flip angle = 90°, spatial resolution = 2.375 × 2.375 mm, slice thickness = 4.0 mm, slices: 24, axial orientation.

Supporting Material II: MRI Imaging Processing and ROI segmentation:

For each MRI sequence, N4ITK MRI bias correction and rotation to volume plane were done. We perform N4ITK MRI bias correction with N4 algorithm in 3D Slicer software (version 4.10.2, www.slicer.org). This algorithm is based on the ITK filters contributed in the following publication: Tustison N, Gee J "N4ITK: Nick's N3 ITK Implementation For MRI Bias Field Correction" (which is introduced in detail at http://hdl.handle.net/10380/3053), and the rotation to volume plane was performed using classical module in 3D Slicer software (version 4.10.2, www.slicer.org).

Since the intensity values of MR images distribute widely, we used z-score normalization to make the image intensities have the properties of a standard normal distribution with $\mu = 1$ and $\sigma = 0$, where μ is the mean value of the intensities, and σ is the standard deviation. The normalized values (also called z scores) of the image intensities (x) were calculated as follows:

$$z = \frac{x - \mu}{\sigma}$$

The segmentation module in the 3D Slicer software (version 4.10.2, www.slicer.org) did the preliminary semi-automatic segmentation according to intensity threshold segmentation. The module is meant to create easy and effecient segmentations on high slice resolution medical images. It can calculate subtraction maps, register images, normalize images, create 3D volumetric ROIs using Delaunay Triangulation, and finally threshold intensities within an ROI. Then the manual corrections such as relabeling and holes filling were done by two professional radiologists with more than ten years of experience in consensus.

Supporting Material III: Feature Extraction:

Before features extraction, the voxel size of each sequence was resampled to 1 × 1 × 1 mm3 and the bin width of gray-level histogram was fixed as 25. After z-score normalization of image pixel intensities, a total of 1408 quantitative imaging features including 13 shape based features, 18 first order statistical features, 75 textural features from original images and 1302 derived features (744 features of Gabor-bank wavelet filtered images and 558 features of Law’s filtered images), were

extracted respectively for T2 images, ADC maps and T1-DCE images using corresponding ROIs.

(1) Shape based features

In this group of features, we included descriptors of the three-dimensional shape and size of the tumor region. Let in the following definitions V denote the volume and A the surface area of the volume of interest. We determined the following shape and size based features:

1. **Compactness 1**= $\frac{V}{\sqrt{\pi}A^{\frac{2}{3}}}$
2. **Compactness 2**= $36\pi\frac{V^2}{A^3}$
3. **Maximum 3d diameter**: The maximum three-dimensional tumor diameter is measured as the largest pairwise Euclidean distance, between voxels on the surface of the tumor volume.
4. **Spherical disproportion** = $\frac{A}{4\pi R^2}$
5. **Sphericity** = $\frac{\pi^{\frac{1}{3}}(6V)^{\frac{2}{3}}}{A}$
6. **Surface area**: The surface area is calculated by triangulation (i.e. dividing the surface into connected triangles) and is defined as:

   $$A=\sum_{\mathrm{i}=1}^{N}\frac{1}{2}\left|a_ib_i \times a_ic_i\right|$$

   Where N is the total number of triangles covering the surface and a, b and c are edge vectors of the triangles.
7. **Surface to volume ratio** = $\frac{A}{V}$
8. **Volume**: The volume (V) of the tumor is determined by counting the number of pixels in the tumor region and multiplying this value by the voxel size.

(2) First order statistical features

The following 17 statistical features were extracted.

Let **X** be the three dimensional image matrix with *N* voxels of the ROI and P be the first order histogram distribution with N_g discrete intensity levels.

1. **IntensityMax:** The maximum intensity value of **X.**
2. **IntensityMin:** The minimum intensity value of **X**.
3. **Median:** The median intensity value of **X**.
4. **IntensityStd:**

$$IntensityStd = (\frac{1}{L*W*H-1}\sum_{i=1}^{L}\sum_{j=1}^{W}\sum_{k=1}^{H}(X(\text{i, j, k}) - IntensityAve)^2)^{1/2}$$

5. **Mean:**

$$\frac{1}{N}\sum_{i}^{N}X(i)$$

6. **Variance:**

$$\sqrt{\sum_{j=1}^{N_g}P(\text{j})*(\text{j}-\sum_{i=1}^{N_g}P(\text{i})*\text{i})^2}$$

7. **Skewness:**

$$\sqrt{\sum_{j=1}^{N_g}P(\text{j})*(\text{j}-\sum_{i=1}^{N_g}P(\text{i})*\text{i})^3}$$

8. **Kurtosis:**

$$\sqrt{\sum_{j=1}^{N_g}P(\text{j})*(\text{j}-\sum_{i=1}^{N_g}P(\text{i})*\text{i})^4}$$

9. **Range:**

   The range of intensity values of **X**.

10. **Mean absolute deviation:**

    The mean of the absolute deviations of all voxel intensities around the mean intensity value

11. **Energy:**

$$\sum_{i}^{N}X(i)^2$$

12. **Entropy:**

$$\sum_{i=1}^{N_g} P(i)\log_2 P(i)$$

13. Entropy_p:

$$\sum_{i=1}^{N_g} \frac{P(i)}{N}\log_2 \frac{P(i)}{N}$$

14. Root mean square:

$$\sqrt{\frac{\sum_i^N X(i)^2}{N}}$$

15. Uniformity:

$$\sum_{i=1}^{N_g} P(i)^2$$

16. **Uniformity_p:**

$$\sum_{i=1}^{N_g} (\frac{P(i)}{N})^2$$

17. Mass:

The sum intensity value of X.

(3) Textural features

Second order statistic texture features, and higher order statistic texture features were extracted. Forty-four second order statistic texture features could be calculated from the Gray Level Co-occurrence Matrix (GLCM). Forty-six high order statistic texture features were calculated from the Gray Level Size Zone Matrix (GLSZM), Gray Level Run Length Matrix (GLRLM), and Neighborhood Gray Tone Difference Matrix (NGTDM). All of the GLCM, GLSZM, GLRLM, and NGTDM based texture feature were calculated using a 2D analysis and then averaged for all slices within the three-dimensional tumor volume.

Gray-Level Co-Occurrence Matrix based features (GLCM)

GLCM based features were second-order statistical texture features, which are defined as a matrix M (i, j; δ, θ) to indicate the relative frequency with intensity

values of pixels (i and j) at the distance of δ in direction θ.

Let:

$M(i, j)$ be the co-occurrence matrix for an arbitrary δ and θ, set $\delta=1$ *and* $\theta=0$ *and 45*

N_g be the number of discrete intensity levels in the images, set as 25,

μ be the mean of $M(i, j)$,

$m_x(\mathrm{i}) = \sum_{j=1}^{N_g} M(\mathrm{i, j})$ be the marginal row probabilities,

$m_y(\mathrm{i}) = \sum_{i=1}^{N_g} M(\mathrm{i, j})$ be the marginal column probabilities, and u_y ,$\mu_{x,}$ be the mean of m_x .and m_y

$HX = -\sum_{i=1}^{N_g} m_x(i)\log(m_x(i),$

$HY = -\sum_{i=1}^{N_g} m_y(i)\log(m_y(i),$

$HXY = -\sum_{i=1}^{N_g}\sum_{j=1}^{N_g} m(i,j)\log(m(i,j)),$

$HXY1 = -\sum_{i=1}^{N_g}\sum_{j=1}^{N_g} m(i,j)\log(m_x(i)m_y(j)).$

$HXY2 = -\sum_{i=1}^{N_g}\sum_{j=1}^{N_g} m_x(i)m_y(j)\log(m_x(i)m_y(j)).$

1. **Energy:**

$$\sum_{i=1}^{N_g}\sum_{j=1}^{N_g}[\mathrm{M(i, j)}]^2$$

2. **Contrast:**

$$\sum_{i=1}^{N_g}\sum_{j=1}^{N_g}(\mathrm{i-j})^2 * M(\mathrm{i, j})$$

3. **Entropy:**

$$\sum_{i=1}^{N_g}\sum_{j=1}^{N_g} M(\mathrm{i, j}) * \log_2 M(\mathrm{i, j})$$

4. **Homogeneity 1:**

$$\sum_{i=1}^{N_g}\sum_{j=1}^{N_g}\frac{M(\mathrm{i, j})}{1+|\mathrm{i-j}|}$$

5. Homogeneity 2:

$$\sum_{i=1}^{N_g}\sum_{j=1}^{N_g}\frac{M(\mathrm{i,j})}{1+|\mathrm{i}-\mathrm{j}|^2}$$

6. Correlation:

$$\frac{\sum_{i=1}^{N_g}\sum_{j=1}^{N_g} ijM(\mathrm{i,j})-\mu_i(\mathrm{i})\mu_j(\mathrm{j})}{\sigma_x(\mathrm{i})\sigma_y(\mathrm{j})}$$

7. Variance:

$$\sum_{i=1}^{N_g}\sum_{j=1}^{N_g}(\mathrm{i}-\mu)^2 M(\mathrm{i,j})$$

8. Sum Average:

$$\sum_{i=2}^{2N_g} iM_{x+y}(\mathrm{i})$$

9. Sum Entropy:

$$-\sum_{i=2}^{2N_g} M_{x+y}(\mathrm{i})\log_2\left[M_{x+y}(\mathrm{i})\right]$$

10. Dissimilarity:

$$\sum_{i=1}^{N_g}\sum_{j=1}^{N_g}|\mathrm{i}-\mathrm{j}|\,\mathrm{M(i,j)}$$

11. Inverse Difference Moment:

$$\sum_{i=1}^{N_g}\sum_{j=1}^{N_g}\frac{\mathrm{M(i,j)}}{1+(\frac{|\mathrm{i}-\mathrm{j}|^2}{N^2})}$$

12. Autocorrelation:

$$\sum_{i=1}^{N_g}\sum_{j=1}^{N_g} ijM(\mathrm{i,j})$$

13. Cluster Prominence

$$\sum_{i=1}^{N_g}\sum_{j=1}^{N_g}\left[i+j-u_x-u_y\right]^4 M(\mathrm{i,j})$$

14. Cluster Shade

$$\sum_{i=1}^{N_g}\sum_{j=1}^{N_g}\left[i+j-u_x-u_y\right]^3 M(\mathrm{i},\mathrm{j})$$

15. Cluster Tendency

$$\sum_{i=1}^{N_g}\sum_{j=1}^{N_g}\left[i+j-u_x-u_y\right]^2 M(\mathrm{i},\mathrm{j})$$

16. Difference Entropy

$$-\sum_{i=0}^{N_g-1} M_{x-y}(i)\log_2\left[M_{x-y}(i)\right]$$

17. Maximum Probability:

$$\max\left\{M(i,j)\right\}$$

18. Sum variance

$$\sum_{i=2}^{2N_g}(i-SE)^2 M_{x+y}(i)$$

19. Informational measure of correlation 1 (IMC1):

$$\frac{HXY-HXY1}{\max\left\{HX-HY\right\}}$$

20. Informational measure of correlation 2 (IMC2):

$$\sqrt{1-\mathrm{e}^{-2(HXY2-HXY)}}$$

21. Inverse Difference Moment Normalized (IDMN):

$$\sum_{i=1}^{N_g}\sum_{j=1}^{N_g}\frac{M(i,j)}{1+(\frac{\left|i-j\right|^2}{N^2})}$$

22. Inverse Difference Normalized (IDN):

$$\sum_{i=1}^{N_g}\sum_{j=1}^{N_g}\frac{M(i,j)}{1+(\frac{\left|i-j\right|}{N^2})}$$

Gray Level Run Length Matrix based features (GLRLM)

GLRLM based features were high-order statistical texture feature, which were defined as $P(i,\ j;\ \theta)$ to indicate the number of times j and gray level i appear

consecutively in the direction θ.

Let:

$P(i, j; \theta)$ be the run-length matrix P for a direction θ, set θ*=0 and 45*

N_g be the number of discrete intensity values,

N_r be the number of different run lengths, and

N_p be the number of voxels in the ROI.

1. **Short Run Emphasis (SRE):**

$$\frac{\sum_{i=1}^{N_g}\sum_{j=1}^{N_r}[\frac{P(\mathrm{i},\mathrm{j};\theta)}{j^2}]}{\sum_{i=1}^{N_g}\sum_{j=1}^{N_r}P(\mathrm{i},\mathrm{j};\theta)}$$

2. **Long Run Emphasis (LRE):**

$$\frac{\sum_{i=1}^{N_g}\sum_{j=1}^{N_r}j^2P(\mathrm{i},\mathrm{j};\theta)}{\sum_{i=1}^{N_g}\sum_{j=1}^{N_r}P(\mathrm{i},\mathrm{j};\theta)}$$

3. **Gray-Level Nonuniformity (GLN):**

$$\frac{\sum_{i=1}^{N_g}[\sum_{j=1}^{N_r}P(\mathrm{i},\mathrm{j};\theta)]^2}{\sum_{i=1}^{N_g}\sum_{j=1}^{N_r}P(\mathrm{i},\mathrm{j};\theta)}$$

4. **Run-Length Nonuniformity (RLN):**

$$\frac{\sum_{j=1}^{N_r}[\sum_{i=1}^{N_g}P(\mathrm{i},\mathrm{j};\theta)]^2}{\sum_{i=1}^{N_g}\sum_{j=1}^{N_r}P(\mathrm{i},\mathrm{j};\theta)}$$

5. **Run Percentage (RP):**

$$\sum_{i=1}^{N_g}\sum_{j=1}^{N_r}\frac{P(\mathrm{i},\mathrm{j};\theta)}{N_p}$$

6. **Low Gray-Level Run Emphasis (LGRE):**

$$\frac{\sum_{i=1}^{N_g}\sum_{j=1}^{N_r}[\frac{P(\mathrm{i},\mathrm{j};\theta)}{i^2}]}{\sum_{i=1}^{N_g}\sum_{j=1}^{N_r}P(\mathrm{i},\mathrm{j};\theta)}$$

7. High Gray-Level Run Emphasis (HGRE):

$$\frac{\sum_{i=1}^{N_g}\sum_{j=1}^{N_r}i^2P(\mathrm{i},\mathrm{j};\theta)}{\sum_{i=1}^{N_g}\sum_{j=1}^{N_r}P(\mathrm{i},\mathrm{j};\theta)}$$

8. Short Run Low Gray-Level Emphasis (SRLGE):

$$\frac{\sum_{i=1}^{N_g}\sum_{j=1}^{N_r}\frac{P(\mathrm{i},\mathrm{j};\theta)}{i^2j^2}}{\sum_{i=1}^{N_g}\sum_{j=1}^{N_r}P(\mathrm{i},\mathrm{j};\theta)}$$

9. Short Run High Gray-Level Emphasis (SRHGE):

$$\frac{\sum_{i=1}^{N_g}\sum_{j=1}^{N_r}\frac{i^2P(\mathrm{i},\mathrm{j};\theta)}{j^2}}{\sum_{i=1}^{N_g}\sum_{j=1}^{N_r}P(\mathrm{i},\mathrm{j};\theta)}$$

10. Long Run Low Gray-Level Emphasis (LRLGE):

$$\frac{\sum_{i=1}^{N_g}\sum_{j=1}^{N_r}\frac{j^2P(\mathrm{i},\mathrm{j};\theta)}{i^2}}{\sum_{i=1}^{N_g}\sum_{j=1}^{N_r}P(\mathrm{i},\mathrm{j};\theta)}$$

11. Long Run High Gray-Level Emphasis (LRHGE):

$$\frac{\sum_{i=1}^{N_g}\sum_{j=1}^{N_r}i^2j^2P(\mathrm{i},\mathrm{j};\theta)}{\sum_{i=1}^{N_g}\sum_{j=1}^{N_r}P(\mathrm{i},\mathrm{j};\theta)}$$

12. Mean:

$$\frac{1}{2N_{\mathrm{g}}}\sum_{i=1}^{N_g}\sum_{j=1}^{N_g}[P(\mathrm{i},\mathrm{j})]^2$$

13. Entropy:

$$\sum_{i=1}^{N_g}\sum_{j=1}^{N_g} P(\mathrm{i, j}) * \log_2 P(\mathrm{i, j})$$

14. Energy:

$$\sum_{i=1}^{N_g}\sum_{j=1}^{N_g} [P(\mathrm{i, j})]^2$$

Gray Level Size Zone Matrix based features (GLSZM)

GLSZM based features were high-order statistical texture features, which were defined as $P(i, j)$ to indicate the areas of size j and gray level i.

Let:

$P(i, j)$ be the size zone of matrix P,

N_g be the number of discrete intensity values,

N_r be the number of different areas sizes,

N_p be the number of voxels in the ROI.

1. **Small Zone Emphasis (SZE):**

$$\frac{\sum_{i=1}^{N_g}\sum_{j=1}^{N_r} [\frac{P(\mathrm{i, j})}{j^2}]}{\sum_{i=1}^{N_g}\sum_{j=1}^{N_r} P(\mathrm{i, j})}$$

2. **Large Zone Emphasis (LZE):**

$$\frac{\sum_{i=1}^{N_g}\sum_{j=1}^{N_r} j^2 P(\mathrm{i, j})}{\sum_{i=1}^{N_g}\sum_{j=1}^{N_r} P(\mathrm{i, j})}$$

3. **Gray-Level Nonuniformity (GLN):**

$$\frac{\sum_{i=1}^{N_g} [\sum_{j=1}^{N_r} P(\mathrm{i, j})]^2}{\sum_{i=1}^{N_g}\sum_{j=1}^{N_r} P(\mathrm{i, j})}$$

4. **Zone-Size Nonuniformity (ZSN):**

$$\frac{\sum_{j=1}^{N_r}[\sum_{i=1}^{N_g}P(\mathrm{i},\mathrm{j})]^2}{\sum_{i=1}^{N_g}\sum_{j=1}^{N_r}P(\mathrm{i},\mathrm{j})}$$

5. Zone Percentage (ZP):

$$\sum_{i=1}^{N_g}\sum_{j=1}^{N_r}\frac{P(\mathrm{i},\mathrm{j})}{N_p}$$

6. Low Gray-Level Zone Emphasis (LGZE):

$$\frac{\sum_{i=1}^{N_g}\sum_{j=1}^{N_r}[\frac{P(\mathrm{i},\mathrm{j})}{i^2}]}{\sum_{i=1}^{N_g}\sum_{j=1}^{N_r}P(\mathrm{i},\mathrm{j})}$$

7. High Gray-Level Zone Emphasis (HGZE):

$$\frac{\sum_{i=1}^{N_g}\sum_{j=1}^{N_r}i^2P(\mathrm{i},\mathrm{j})}{\sum_{i=1}^{N_g}\sum_{j=1}^{N_r}P(\mathrm{i},\mathrm{j})}$$

8. Small Zone Low Gray-Level Emphasis (SZLGE):

$$\frac{\sum_{i=1}^{N_g}\sum_{j=1}^{N_r}\frac{P(\mathrm{i},\mathrm{j})}{i^2 j^2}}{\sum_{i=1}^{N_g}\sum_{j=1}^{N_r}P(\mathrm{i},\mathrm{j})}$$

9. Small Zone High Gray-Level Emphasis (SZHGE):

$$\frac{\sum_{i=1}^{N_g}\sum_{j=1}^{N_r}\frac{i^2P(\mathrm{i},\mathrm{j})}{j^2}}{\sum_{i=1}^{N_g}\sum_{j=1}^{N_r}P(\mathrm{i},\mathrm{j})}$$

10. Large Zone Low Gray-Level Emphasis (LZLGE):

$$\frac{\sum_{i=1}^{N_g}\sum_{j=1}^{N_r}\frac{j^2P(\mathrm{i},\mathrm{j})}{i^2}}{\sum_{i=1}^{N_g}\sum_{j=1}^{N_r}P(\mathrm{i},\mathrm{j})}$$

11. Large Zone High Gray-Level Emphasis (LZHGE):

$$\frac{\sum_{i=1}^{N_g}\sum_{j=1}^{N_r} i^2 j^2 P(\mathrm{i, j})}{\sum_{i=1}^{N_g}\sum_{j=1}^{N_r} P(\mathrm{i, j})}$$

12. Gray-Level Variance (GLV):

$$\sum_{i=1}^{N_g}\sum_{j=1}^{N_r}\{\mathrm{iP(i, j)} - \frac{\sum_{i=1}^{N_g} i[\sum_{j=1}^{N_r} P(\mathrm{i, j})]}{N_g N_r}\}$$

13. Zone-Size Variance (ZSV):

$$\sum_{i=1}^{N_g}\sum_{j=1}^{N_r}\{\mathrm{jP(i, j)} - \frac{\sum_{i=1}^{N_g} i[\sum_{j=1}^{N_r} P(\mathrm{i, j})]}{N_g N_r}\}$$

Neighborhood Gray Tone Difference Matrix based features (NGTDM)

NGTDM based features were high-order statistical texture features, which were defined as *S(i)* to indicate the sum of the absolute value between gray intensity level i and it's neighbors' average intensity.

Let:

S(i) be the sum of absolute value between gray intensity level i and its neighbors' average intensity,

C(i) be the number of voxels with the gray intensity level I,

N_g be the number of discrete intensity values.

1. **Coarseness:**

$$\frac{1}{\varepsilon + \sum_{i=1}^{N_g} \frac{C(\mathrm{i})\,\mathrm{S(i)}}{\sum_{i=1}^{N_g} C(\mathrm{i})}}$$

2. **Contrast:**

$$\frac{\sum_{i=1}^{N_g}\sum_{j=1}^{N_g} C(\mathrm{i})\,\mathrm{C(j)(i-j)^2}}{(\sum_{i=1}^{N_g} C(\mathrm{i}))^2} * \sum_{i=1}^{N_g} S(\mathrm{i}) * \frac{1}{N_g(\mathrm{N}_g - 1)\sum_{i=1}^{N_g} C(\mathrm{i})}$$

3. **Busyness:**

$$\frac{\sum_{i=1}^{N_g} C(\mathrm{i})\mathrm{S}(\mathrm{i})}{\sum_{i=1}^{N_g}\sum_{j=1}^{N_g}(\mathrm{iC}(\mathrm{i})-\mathrm{jC}(\mathrm{j}))}, \quad C(\mathrm{i})\neq 0, \mathrm{C}(\mathrm{j})\neq 0$$

4. **Complexity:**

$$\frac{1}{(\sum_{i=1}^{N_g} C(\mathrm{i}))^2} * \sum_{i=1}^{N_g}\sum_{j=1}^{N_g}\frac{|\mathrm{i}-\mathrm{j}|(\mathrm{C}(\mathrm{i})\mathrm{S}(\mathrm{i})+\mathrm{C}(\mathrm{j})\mathrm{S}(\mathrm{j}))}{(\mathrm{C}(\mathrm{i})+\mathrm{C}(\mathrm{j}))}, \quad C(\mathrm{i})\neq 0, \mathrm{C}(\mathrm{j})\neq 0$$

5. **Strength:**

$$\frac{\sum_{i=1}^{N_g}\sum_{j=1}^{N_g}(\mathrm{C}(\mathrm{i})-\mathrm{C}(\mathrm{j}))*(\mathrm{i}-\mathrm{j})^2}{\sum_{i=1}^{N_g} C(\mathrm{i}) * \sum_{i=1}^{N_g} S(\mathrm{i})}, \quad C(\mathrm{i})\neq 0, \mathrm{C}(\mathrm{j})\neq 0$$

(4) Wavelet features: first order statistical and texture features of a wavelet filtered image.

A total of 1302 derived features were extracted for each sequence, with the Gaussian filter and a wavelet-based filter. These features were computed on the filtered images. The original image was filtered by 8 filters. For each image, the first order statistical and texture features were computed. Finally, 3906 wavelet-based features were extracted.

Supporting Material IV: Radiomics Feature normalization:

The z-score normalization process of each feature includes two steps: for one feature, the mean value was subtracted, and then the standard deviation was divided by the result, finally, all the features had a mean value of 0 and a standard deviation of 1. The calculating formula of feature normalization is as follows:

$$\mathrm{z} = \frac{\mathrm{x}-\mu}{\sigma}$$
